# Marine Natural Products as Breast Cancer Resistance Protein Inhibitors

**DOI:** 10.3390/md13042010

**Published:** 2015-04-03

**Authors:** Lilia Cherigo, Dioxelis Lopez, Sergio Martinez-Luis

**Affiliations:** 1 Department of Organic Chemistry, Chemistry School, Faculty of Natural Sciences, Exact and Technology, University of Panama, Panama City P.O. Box 3366, Panama; E-Mail: chery826@hotmail.com; 2 Department of Biotechnology, Acharya Nagarjuna University, Nagarjuna Nagar, Guntur 522510, India; E-Mail: dioxelis.lopez@indicasat.org.pa; 3 Center for Drug Discovery and Biodiversity, Institute for Scientific Research and Technology Services (INDICASAT), Clayton, Panama City P.O. Box 0843-01103, Panama

**Keywords:** marine natural products, ABC transporters, breast cancer resistance protein (BCRP), multidrug resistance (MDR)

## Abstract

Breast cancer resistance protein (BCRP) is a protein belonging to the ATP-binding cassette (ABC) transporter superfamily that has clinical relevance due to its multi-drug resistance properties in cancer. BCRP can be associated with clinical cancer drug resistance, in particular acute myelogenous or acute lymphocytic leukemias. The overexpression of BCRP contributes to the resistance of several chemotherapeutic drugs, such as topotecan, methotrexate, mitoxantrone, doxorubicin and daunorubicin. The Food and Drugs Administration has already recognized that BCRP is clinically one of the most important drug transporters, mainly because it leads to a reduction of clinical efficacy of various anticancer drugs through its ATP-dependent drug efflux pump function as well as its apparent participation in drug resistance. This review article aims to summarize the different research findings on marine natural products with BCRP inhibiting activity. In this sense, the potential modulation of physiological targets of BCRP by natural or synthetic compounds offers a great possibility for the discovery of new drugs and valuable research tools to recognize the function of the complex ABC-transporters.

## 1. Introduction

### 1.1. Breast Cancer Resistance Protein

**Figure 1:**
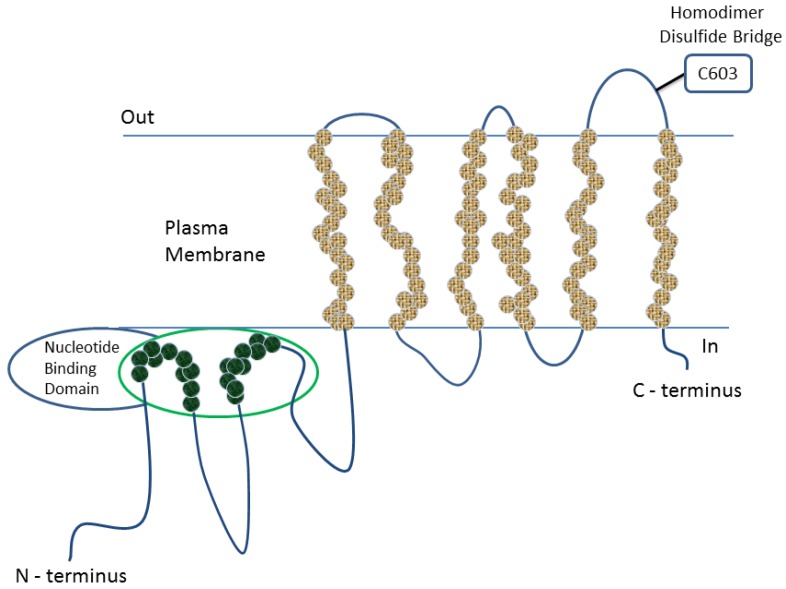
Structure of breast cancer resistance protein (BCRP).

Breast cancer resistance protein (BCRP/ABCG2/MXR/ABCP) is an ATP-dependent efflux transporter, which belongs to the large ATP-binding cassette (ABC) transporter family present on cell membranes, and it is classified into the G subfamily of these transporters [1,2,3,4,5,6]. BCRP, which is a transmembrane protein encoded by the ABCG2 gene, was originally isolated from adriamycin-resistant breast cancer cell lines (MCF-7/AdrVp) [7]. Almost simultaneously, BCRP cDNA sequences were also cloned from mitoxantrone-resistant human cancer cell lines (MXR) [8] and human placenta (ABCP) [9]. BCRP is composed of 655 amino acids (72-kDa) and organized into six transmembrane α-helices, containing only one nucleotide-binding domain (NBD) near its *N*-terminal and one membrane-spanning domain (MSD) (Figure 1) [1,2,3,4,5,6]. It is one of the smallest human ABC proteins reported so far, which *per se* is a half transporter that becomes a functional efflux pump when a disulfide bridge at Cys 603 of two proteins is homodimerized. It is important to note that although the minimal functional unit of this transporter is a dimer, higher oligomeric forms (up to homododecamers) have also been reported [1,2,3,4,5,6].

### 1.2. Functions of BCRP

BCRP works as an efflux transporter for unwanted substances at the plasma membrane of many cells in normal tissues such as placenta, brain, prostate, small intestine, testes, ovaries, liver, adrenal gland, uterus and the central nervous system [1,2,3,4,5,6]. BCRP is expressed all over the body, but it expresses at a higher frequency in the placenta, which suggests that BCRP plays a role in protecting the fetus by preventing potentially harmful substances from entering the uterus [10]. BCRP is found in the apical membrane of epithelial cells, intestines, kidneys, placenta and the blood-brain barrier. It is well know that BCRP restricts drug accumulation in the central nervous system [1,11]. In summary, physiological distribution, including the presence of BCRP on cell barriers, reveals its important role in cellular protection against toxic substances [1,2,3,4,5,6,10].

### 1.3. Importance in Therapy

BCRP may actively pump substances out of the cells affecting the absorption, distribution and secretion of several drugs and endogenous substrates such as estrogens, folic acid and protoporphyrin. Among the therapeutic drugs, which are substrates of BCRP are antibiotics, antivirals, chemotherapeutic agents, HMG-CoA reductase inhibitors, steroids and phytoestrogens [1,2,3,4,5]. The Food and Drugs Administration (FDA) has already recognized that BCRP is clinically one of the most important drug transporters, mainly because it is well known that this protein plays an important role in drug-drug interactions in humans as well that it participates in drug resistance [12]. ABC transporter proteins are fundamental molecules in the multidrug-resistant phenotype of cancer cells, in particular acute myelogenus or acute lymphocytic leukemias [1,2,3,4]. The overexpression of BCRP is involved in the resistance to several chemotherapeutic drugs, such as topotecan, methotrexate, mitoxantrone, doxorubicin and daunorubicin [1,2,3,4,5]. This indeed shows that BCRP could reduce clinical efficacy of several anticancer drugs, and this will be an important tool to success in cancer treatment [9].

### 1.4. BCRP Inhibitors

It has been shown that the number of compounds described as inhibitors of BCRP as well as their structural diversity is large (Table 1). Recently it has been established that some of BCRP substrates are also substrates for P glycoprotein (P-gp). This information has been used to develop specific and non-competitive inhibitors for BCRP. [1,13].

**Table 1:** Selected examples of classical BCRP inhibitors.

Inhibitor	Substrate	Cell Line	ATPase Activity	Photoaffinity Labeling	Specificity
FTC [14,15]	MX	S1-M1-3.2	Inhibited	XX	Yes
Novobiocin [16]	TPT	PC6/SN2-5H2	XX	XX	Yes
Elacridar [17,18,19]	MX	MCF-7 MX	Inhibited	Unaffected	No
Reserpine [20,21]	H33342	SP	XX	Inhibited	No
Cyclosporin A [22,23,24]	PhA, MX	HEK/482R	Inhibited	Unaffected	No
Tariquidar [25]	MX	H460/MX20	Stimulated	XX	No
Ortataxel [26]	MX	8226/MR20	Decreased	XX	No
Gefitinib [27]	H33342	PLB-ABCG2	Inhibited ^a^	XX	No

MX: Mitoxantrone; PhA: Pheophorbide A; TPT: Topotecan; H33342: Hoechst 33342 dye; ^a^: At high concentration; XX: Not yet reported.

### 1.5. In Silico Studies with BCRP Inhibitors

Based on the clinical importance of BCRP in drug resistance, its use as a target for the development of new effective molecules, which may possess improved pharmacokinetics parameters, better levels of efficacy and be safer, is unquestionable. Therefore, application of *in silico* models could be an alternative for obtaining valuable information that allows the development of more specific BCRP inhibitors based on the marine inhibitors so far described.

From its assessment in drug discovery, *in silico* prediction models have allowed the detection and selection of promising molecules from libraries or databases [28,29]. Moreover, these models also provide information regarding the possible mechanisms of protein-ligand interactions [30].

A useful tool for *in silico* prediction is the existence of a high-resolution structures of proteins because it allows to predict the structures and physicochemical characteristics of the complex formed between a specific protein and its ligands. Unfortunately, the high-resolution structure of BCRP is still not available. Currently, there are only models of the BCRP structure, based on the crystalline structures of related proteins such as the transporter Sav1866 from *Staphylococcus aureus* [31,32,33] and the lipid flippase MsbA from *Vibrio cholerae* (VC-MsbA) [33,34]. These models predict the BCRP topology based on theoretical computer calculations and they are consistent with several experimental features, for instance, the presence of multidrug sites in a large central cavity binding [31,32,34]. Predicted structures can be used to perform docking analysis and/or the interpretation of some biochemical parameter. However, it is important to highlight that the obtained results may be unreliable for drug design and screening.

**Figure 2:**
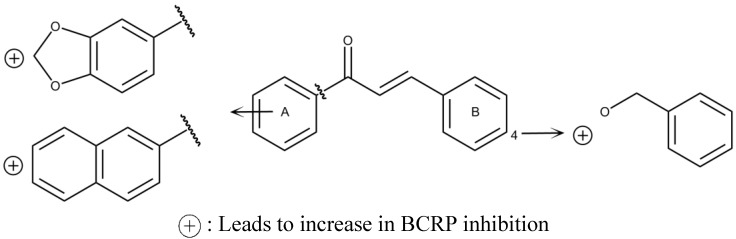
Structure-activity relationship of chalcones for BCRP inhibition.

**Figure 3:**
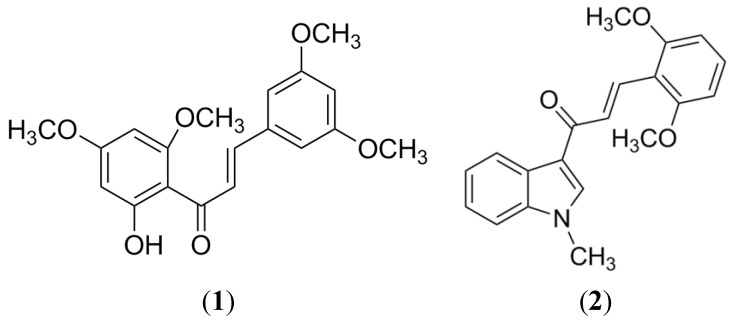
Structure of the most promising chalcone derivatives as BCRP inhibitors.

On the other hand, there are methods based on structural similarity of ligands for known substrates, which usually give more accurate comparisons than those based on the protein structure. One example is the structure-activity relationship analysis, SAR and QSAR, focused on establishing a correlation between descriptors that represent information of molecular structures of ligands and their biological activities [35,36,37]. With these models it is possible to detect the specific substituents or the parts of a molecule (pharmacophore) that are essential or not for a previously recognized biological function [35,36,37]. For instance, the SAR analysis of 54 chalcones with different degrees of substitution and their evaluation in BCRP-transfected HEK293 cells resulted in the identification of a flavonoid-specific inhibitory site, which seems to be polyspecific because when the original phenyl A-ring was replaced by long-chain substituents, its biological effect was not affected. From all evaluated substituents, 2′-naphthyl and 3′, 4′ methylene-dioxy-phenyl were the most effective (Figure 2). Also, the substitution at position 4 of ring B is critical; in this position an *O*-benzyl residue is the most effective substituent for the inhibition and cytotoxicity [38]. Many other studies have demonstrated that chalcones are promising selective BCRP inhibitors [4,39,40,41], such as Compounds **1** and **2** (Figure 3), which have now become good candidates for preclinical experiments [4].

**Figure 4:**
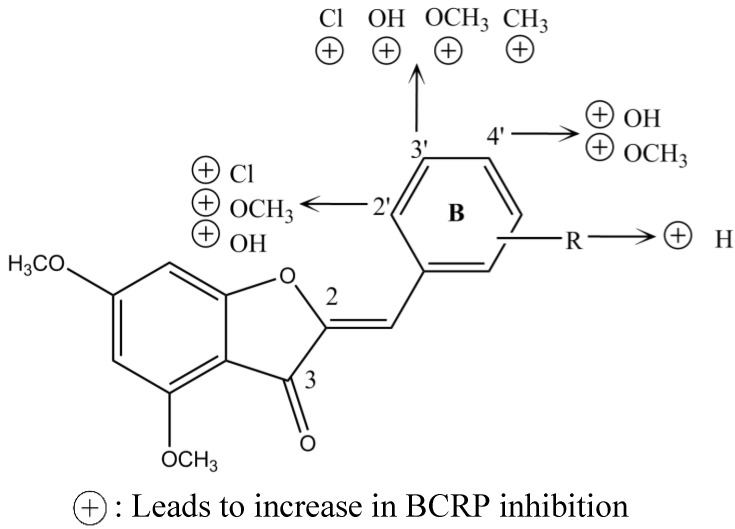
Structure-activity relationship of dimethoxyaurones for BCRP inhibition.

Another study displayed the potential of the 4,6-dimethoxy aurones derivatives as BCRP inhibitors. Aurones were found to re-sensitize BCRP expressing cancer cells to mitoxantrone and showed low antiproliferative activity against cell lines. Structure-activity analysis showed that substitution of ring B had the best P-gp inhibition, with a preference for position 3′ (Figure 4). In contrast, substitution of the benzylidene portion of ring B was less important for the BCRP inhibition, showing a low variation of activity. In conclusion, aurones derivatives with good BCRP inhibitory activity are poor P-gp inhibitors and *vice versa* [42].

**Figure 5:**
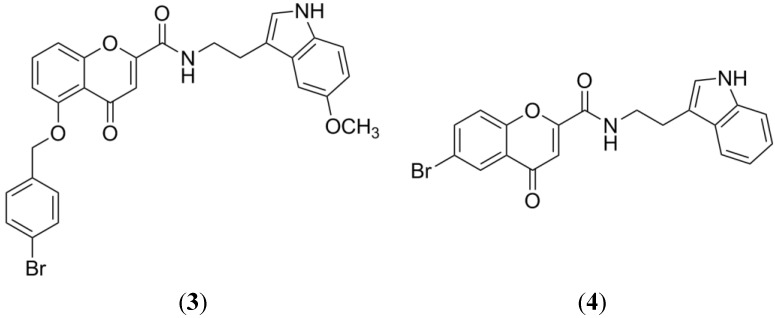
Structure of the most promising chromone derivatives as BCRP inhibitors.

As a final example, we have the chromone type compounds, which have proved to be selective and potent BCRP inhibitors. Boudmendjel, *et al.* synthesized several benzopyrones derivatives in order to perform a SAR study with this type of compounds [43]. His work demonstrated the impact of the hydrophobic chains, the steric hindrance by bulky groups and the role of methoxy groups (lipophilicity) in the inhibition of BCRP function. Furthermore, a QSAR study with 34 chromones showed that hydrophobicity and shape are key physicochemical parameters to improve the BCRP inhibition, while hydrogen bond donor capacity is an unfavorable feature for this activity [44]. The most potent chromones as BCRP inhibitors resulted from coupling 6-substituted-4-oxo-4H-chromene-2-carboxylic acid with tryptamine, leading to MBL-II-141 (**3**) and *N*-(2-(1*H*-Indol-3-yl)ethyl)-6-bromo-4-oxo-4*H*-chromene-2-carboxamide (**4**), (Figure 5) [45,46]. MBL-II-141 led a potent sensitization of ABCG2 positive xenografts to irinotecan through an *in vivo* ABCG2 inhibition [47]. Compound **4** showed potency, selectivity and very low toxicity *in vitro*. Therefore this compound is being considered for *in vivo* evaluation.

## 2. BCRP Inhibitors from Marine Sources

Oceans are a vast reservoir of bioactive natural products, some of which exhibit important and unique biological properties. Thus, many compounds isolated from marine sources are currently used in clinical trials or as prototypes for the design and synthesis of new therapeutic agents [48,49,50].

In the past 30 years, at least seven marine compounds or their derivatives have been approved for clinical use, mainly in cancer therapy, which is the fourth leading cause of death in middle- and high-income countries. Cancer is responsible for more than 8.2 million deaths per year worldwide, and its incidence is increasing, giving an estimate that for the next two decades there will be an annual average of 22 million of new cancer cases [48,51]. Undoubtedly, compounds derived from marine sources have marked a milestone in cancer treatment. However, there are still many obstacles for overcoming this disease, in particular the fact that many types of cancer develop resistance to several important drugs, as well as the devastating side effects that result from their use. For these reasons, it is necessary to continue with the search of novel drugs with more efficacy and safety [52,53].

As part of a series of reviews focused on the description of marine natural products with inhibitory properties on transporters belonging to the ATP-binding cassette (ABC) superfamily [54], in this paper we describe a review of marine natural products and derivatives as BCRP inhibitors. As mentioned earlier, BCRP plays an important role in the drug resistance of cancer treatments, therefore the potential modulation of physiological targets of BCRP by natural or synthetic compounds offers great possibilities for the discovery of new anticancer drugs and valuable research tools for the study of ATP-binding cassette (ABC) transporters complex.

### 2.1. Fumitremorgin C

**Figure 6:**
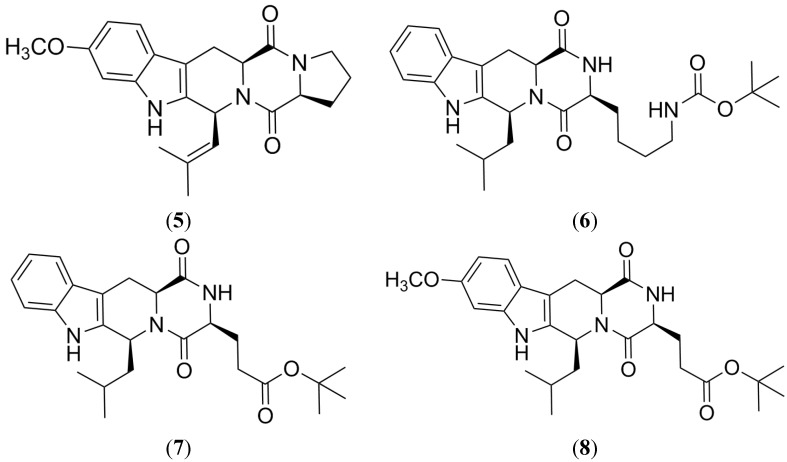
Structure of fumitremorgin (FTC) and related compounds.

Fumitremorgin C (FTC, **5**) is a prenylated indole alkaloid derived from the amino acids l-tryptophan and l-proline, and it was isolated from several strains of both marine and terrestrial fungi [55,56,57,58]. FTC (Figure 6) is the first strong and specific BCRP inhibitor, and its value as a research tool is widely reported. Several studies have proved that **5** reverts resistance to topotecan, mitoxantrone and doxorubicin, on S1-M1-3.2 cells that over express BCRP [59,60,61]. However, FTC induces some severe side effects such as tremors or convulsions in mice and other animals, which are associated with toxicity in the central nervous system [62]. Such results led to the exclusion of this compound from further clinical studies. Its strong specificity and potency was the basis for the design of less toxic related compounds. One of the earliest efforts to detect **5** analogues with BCRP inhibitor properties was the evaluation of a combinatorial panel of 42 indolyl diketopiperazine FTC derivatives, with Ko132 (**6**), Ko134 (**7**) and Ko143 (**8**) [63,64,65] being the most promising leads.

### 2.2. Tryprostatin A

**Figure 7:**
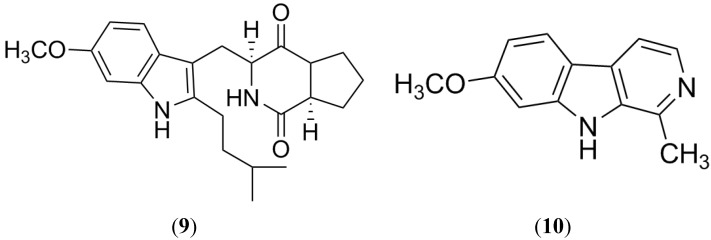
Structure of Tryprostatin A and Harmine.

Tryprostatin A (**9**) is a natural analog of FTC isolated from the marine fungus *Aspergillus fumigatus* BM939 along with tryprostatin B [66,67,68]. Both molecules are formed by the condensation of a proline unit and an isoprenyl tryptophan residue, leading to a diketopiperazine unit. Both compounds stop cell cycle progression of tsFT210 cancer cells in the G2/M phase [67]. In the experiments to reverse a mitoxantrone-resistant phenotype and inhibit the cellular BCRP-dependent mitoxantrone accumulation using the displayed BCRP-mediated MDR cells EPG85-257RNOV (human gastric carcinoma cell line) and MCF-7/AdrVp (human breast cancer cells), only tryprostatin A (Figure 7) showed activity in a concentration range of 10–50 µM and did not exhibit cytotoxicity at evaluated concentrations [69].

### 2.3. Harmine

Harmine (**10**) is a beta-carboline alkaloid with an extensive distribution in nature [37], which is isolated from marine brown alga, some cyanobacteria and marine animals [70,71,72]. Harmine (Figure 7) possesses some biological properties including antimicrobial, antiplasmodial, antifungal, antioxidant, antitumoral, antimutagenic, cytotoxic and hallucinogenic activity [70,71,72].

A study employing MDA-MB-231 cells, a breast cancer cell line that overexpresses BCRP, showed that this alkaloid inhibits BCRP. Harmine significantly decreases resistance to the anticancer drugs such as mitoxantrone and camptothecin mediated by BCRP. The effect of **10** against MDR cells appears to be specific against BCRP because this compound does not show inhibition of P-gp overexpressing cells [73].

Although **10** was efficient as a reversal MDR agent, its neurotoxicity and cytotoxicity have hindered its clinical development, although it constitutes a good lead for the development of BCRP reversal agents [73].

### 2.4. Botryllamides

**Figure 8:**
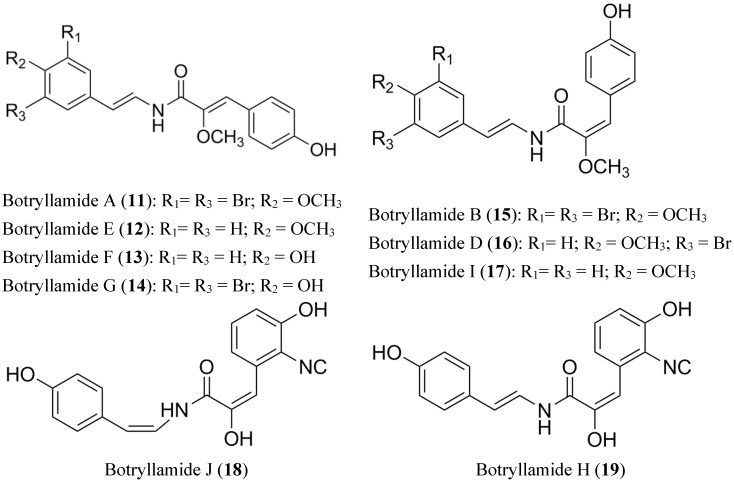
Structure of Botryllamides.

Botryllamides (**11**–**19**) compose a large group of compounds isolated from several species of Ascidian genus *Botryllus* [74,75,76]. These metabolites belong to the first class of marine molecules containing a dehydrotyrosine residue. *Botryllus* extracts showed interesting activity in a screening performed by the National Cancer Institute (NCI) against the most common transporters (BCRP, P-gp and MDR-1) in MDR cells. From these extracts, botryllamides (Figure 8) were identified as the active constituents, and they were shown to be selective BCRP inhibitors. The effect of botryllamides against BCRP was evaluated as their capacity for inhibiting the BCRP-mediated BODIPY-prazosin efflux in BCRP transfected HEK293 cells, competition of [^125^I]-iodoarylazidoprazosin labeling of BCRP and promoting BCRP-associated ATPase activity [77].

**Figure 9:**
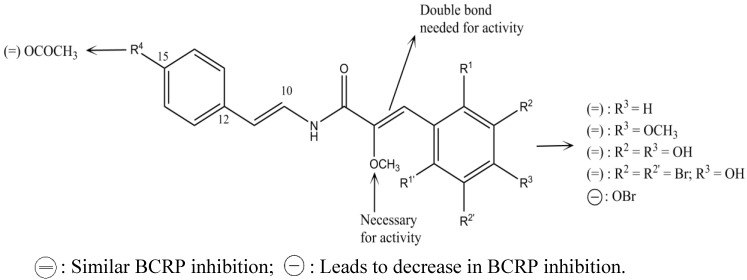
Structure-activity relationship of Botryllamides for BCRP inhibition.

Because of their relatively low cytotoxicity, these compounds could be useful clinically. In addition, a series of botryllamide derivatives, including botryllamide F and G, were achieved in order to establish a possible structural basis for their activity. The BCRP inhibitory activity suggests that the presence of a 2-methoxy-p-coumaric acid moiety conjugated with a double bound is essential; but extended conjugation at C10-C17 (variations in the substituents on the aryl groups) is not crucial (Figure 9). Finally, this SAR study proposed two binding interactions between botryllamides and BCRP [78].

### 2.5. Lamellarin O

Lamellarins are natural compounds isolated from several marine invertebrates (mainly ascidians and sponges) and are made up of an unusual heterocyclic ring system [79,80,81,82,83,84,85,86,87]. Many of these natural products exhibit some biological activities including antitumoral and anti-HIV activity [79,80,81,82,83,84,85,86,87]. According to the substituted pyrrole, these marine alkaloids are classified into three subgroups: Lamellarins characterized by the presence of the pyrrole ring fused with adjacent aromatic rings (forming a pentacyclic core), a central pyrrole ring unfused to adjacent aromatic rings and a quinoline ring instead of pyrrole [87].

**Figure 10:**
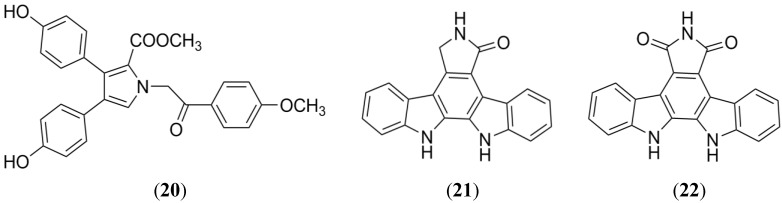
Structure of lamellarin O, K252C and arcyriaflavin A.

Lamellarin O (**20**) isolated from the southern Australian marine sponge *Ianthella* sp. displayed a potent and selective BCRP inhibition. A mitoxantrone efflux assay based on flow cytometry and NCI-H460/ MX20 cells (mitoxantrone resistant human lung cancer) was used to evaluate the inhibitory properties of **20** (Figure 10). Furthermore, SAR analysis and *in silico* studies have established that the pharmacophoric group of **20** is composed of a methoxy-acetophenone, carboxylic ester and phenolic moieties [88].

### 2.6. Indolocarbazole Alkaloids

Indolocarbazole alkaloids are a class of natural compounds, which possess a wide range of biological properties, especially anticancer activity [89,90,91]. From the EtOAc extract of a marine-derived actinomycete strain Z039-2 collected on the coast of Qingdao, China, two indolocarbazole alkaloids (Figure 10) with anticancer properties, K252c (staurosporine aglycon, **21**) and arcyriaflavin A (**22**), were obtained [92]. A group of indolocarbazoles (including **21** and **22**) were evaluated, using a wild-type of BCRP-transfected HEK-293 cells, in order to detect BCRP inhibition. Several of the tested compounds showed important inhibitory activity against the BCRP-mediated efflux of pheophorbide A, **21** and **22** were the most potent. In photoaffinity labeling experiments, K252c and arcyriaflavin A were able to prevent the [^125^I]-iodoarylazidoprazosin labeling of BCRP with IC_50_ values of 0.37 and 0.23 mmoL/L, respectively. K252c and arcyriaflavin A showed low cytotoxicity on BCRP-transfected cells in a sulforhodamine B assay, and reduced the relative resistance to SN-38 (7-ethyl-10-hydroxycamptothecin) [93].

### 2.7. Terrein

**Figure 11:**
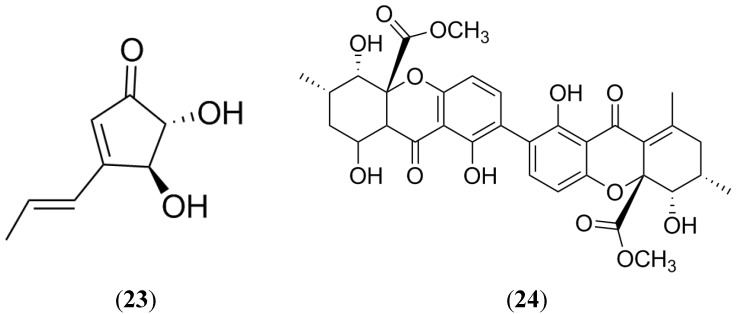
Structure of Terrein and Secalonic acid D (SAD).

Terrein (**23**) is a metabolite isolated from some marine strains of *Aspergillus terreus* (mainly PF-26 and PT06-2) and *Emericella variecolor* [94,95,96]. Terrein (Figure 11) exhibited interesting properties including antibacterial and antiinflammatory activities, as well as melanogenesis, angiogenin secretion and proteasome inhibition properties [95,96,97,98,99,100,101,102]. Regarding its anticancer properties, **23** displayed strong cytotoxicity (100-fold more potent than paclitaxel) against breast (MCF-7), pancreatic (PANC-1) and liver (HepG2) cancer cells. Terrein also showed a significant reduction in BCRP-expressing cells through the activation of the caspase-7 pathway and the inhibition of the Akt signaling pathway [102].

### 2.8. Secalonic Acid D

Secalonic acid D (SAD) is a mycotoxin produced by *Penicillium oxalicum*, which also has been obtained from a lichen marine-derived fungus and other mangrove endophytic fungus [103,104,105,106,107]. SAD (**24**) has been widely investigated for its anticancer properties and it is well known that **24** acts as a DNA topoisomerase I inhibitor. SAD (Figure 11) also displayed significant cytotoxic activity and provoked apoptosis in K562 and HL60 myeloid leukemia cell lines by blocking the G1 phase of the cell cycle in the GSK-3β/β-catenin/c-Myc pathway. Furthermore, **24** is an inhibitor of murine pituitary adenoma GH3 cells in a dose-dependent manner promoting apoptosis [106,107]. Regarding cancer resistance, **24** showed potent cytotoxicity on MDR cells (P-gp-, MRP1- and BCRP-overexpressing multidrug resistance cells) and their parental cells through an MTT assay. In these cells, **24** could down-regulate the expression of BCRP protein by activation of calpain 1. SAD could down-regulate the expression of BCRP and decrease the percentage of side population cells in lung cancer cells. All these findings have proved that **24** is an interesting molecule that possesses a potent cytotoxic activity by a mechanism of inducing BCRP degradation of the activation of calpain 1 [108]. Unfortunately, **24** has a high toxicity, making clinical usage difficult, but it could be considered as a leading compound for the development of new BCRP inhibitors.

### 2.9. Naphthopyrones

**Figure 12:**
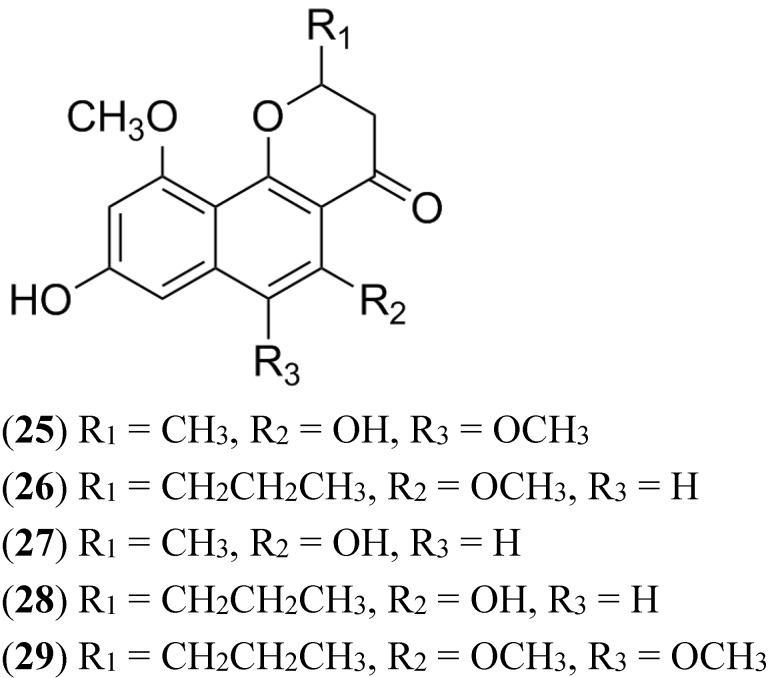
Structure of naphthopyrones actives against BCRP.

Some organic extracts prepared from marine crinoids *Capillaster multiradiatus*, *Comanthus parvicirrus* and a unidentified crinoid of the Comasteridae family, showed activity in a high throughput assay measuring accumulation of the BCRP specific substrate pheophorbide A in BCRP-overexpressing NCI-H460 MX20 cells [109]. Bioassay-guided fractionation of the extracts led to the isolation of five new naphthopyrones along with six known compounds. The isolates were divided into two groups: Linear and angular. This difference in structure was important for the activity against BCRP since only the angular naphthopyrones (**25**–**29**) showed significant activity (ranging from 27% to 59%) [109]. Although active naphthopyrones (Figure 12) showed moderate activity against BCRP- overexpressing cells, it is an example for the role of marine natural products as a reservoir of molecules with inhibitory activity of the MDR transporters.

## 3. Conclusions

BCRP can expel a broad range of structurally different metabolites out of cells. In this context, a very active BCRP transporter could potentially diminish drug delivery to the target organ and lead to treatment resistance, despite peripheral drug concentrations being within their therapeutic range. The development of compounds with BCRP inhibitory properties from marine sources is one of the most important approaches in drug discovery due to the fact that the marine ecosystem has shown a unique chemical diversity. In recent years, several marine compounds with BCRP inhibitor properties have been isolated, most of them belonging to a group of alkaloids. These types of molecules (alkaloids) have shown potent and selective MDR activity against certain cancer cells, but unfortunately they have some toxicity, which has limited their pharmaceutical development. All these agents represent new research tools for the discovery and development of efficient BCRP inhibitors, which may have potential use, *per se* or in combination, for the treatment of cancer and for the design of rational analogues with higher activity, less toxicity and fewer pharmacokinetic interactions.

## References

[1] Lecerf-Schmidt F., Peres B., Valdameri G., Gauthier C., Winter E., Payen L., di Pietro A., Boumendjel A. (2013). ABCG2: Recent discovery of potent and highly selective inhibitors. Future Med. Chem..

[2] Natarajan K., Xie Y., Baer M.R., Ross D.D. (2012). Role of breast cancer resistance protein (BCRP/ABCG2) in cancer drug resistance. Biochem. Pharmacol..

[3] Noguchi K., Katayama K., Sugimoto Y. (2014). Human ABC transporter ABCG2/BCRP expression in chemoresistance: Basic and clinical perspectives for molecular cancer therapeutics. Pharmgenomics Pers. Med..

[4] Mao Q., Unadkat J.D. (2015). Role of the breast cancer resistance protein (BCRP/ABCG2) in drug transport-an update. AAPS J..

[5] Doyle L., Ross D.D. (2003). Multidrug resistance mediated by the breast cancer resistance protein BCRP (ABCG2). Oncogene.

[6] Stacy A.E., Jansson P.J., Richardson D.R. (2013). Molecular pharmacology of ABCG2 and its role in chemoresistance. Mol. Pharmacol..

[7] Doyle L.A., Yang W., Abruzzo L.V., Krogmann T., Gao Y., Rishi A.K., Ross D.D. (1998). A multidrug resistance transporter from human MCF-7 breast cancer cells. Proc. Natl. Acad. Sci. USA.

[8] Miyake K., Mickley L., Litman T., Zhan Z., Robey R., Cristensen B., Brangi M., Greenberger L., Dean M., Fojo T. (1999). Molecular cloning of cDNAs which are highly overexpressed in mitoxantrone-resistant cells: Demonstration of homology to ABC transport genes. Cancer Res..

[9] Allikmets R., Schriml L., Hutchinson A., Romano-Spica V., Dean M. (1998). A human placenta-specific ATP-binding cassette gene (ABCP) on chromosome 4q22 that is involved in multidrug resistance. Cancer Res..

[10] Wang H., Zhou L., Gupta A., Vethanayagam R.R., Zhang Y., Unadkat J.D., Mao Q. (2006). Regulation of BCRP/ABCG2 expression by progesterone and 17 beta-estradiol in human placental BeWo cells. Am. J. Physiol. Endocrinol. Metab..

[11] Loscher W., Potska H. (2005). Drug resistance in brain diseases and the role of drug efflux transporters. Nat. Rev. Neurosci..

[12] König J., Müller F., Fromm M.F. (2013). Transporters and drug-drug interactions: Important determinants of drug disposition and effects. Pharmacol. Rev..

[13] Szakacs G., Paterson J.K., Ludwig J.A., Booth-Genthe C., Gottesman M.M. (2006). Targeting multidrug resistance in cancer. Nat. Rev. Drug Discov..

[14] Robey R.W., Honjo Y., van de Laar A., Miyake K., Regis J.T., Litman T., Bates S.E. (2001). A functional assay for detection of the mitoxantrone resistance protein, MXR (ABCG2). Biochim. Biophys. Acta.

[15] Ozvegy C., Litman T., Szakács G., Nagy Z., Bates S., Váradi A., Sarkadi B. (2001). Functional characterization of the human multidrug transporter, ABCG2, expressed in insect cells. Biochem. Biophys. Res. Commun..

[16] Shiozawa K., Oka M., Soda H., Yoshikawa M., Ikegami Y., Tsurutani J., Nakatomi K., Nakamura Y., Doi S., Kitazaki T. (2004). Reversal of breast cancer resistance protein (BCRP/ABCG2)-mediated drug resistance by novobiocin, a coumermycin antibiotic. Int. J. Cancer.

[17] Pick A., Müller H., Wiese M. (2008). Structure-activity relationships of new inhibitors of breast cancer resistance protein (ABCG2). Bioorg. Med. Chem..

[18] Ahmed-Belkacem A., Pozza A., Muñoz-Martínez F., Bates S.E., Castanys S., Gamarro F., di Pietro A., Pérez-Victoria J.M. (2005). Flavonoid structure-activity studies identify 6-prenylchrysin and tectochrysin as potent and specific inhibitors of breast cancer resistance protein ABCG2. Cancer Res..

[19] Pozza A., Perez-Victoria J.M., Sardo A., Ahmed-Belkacem A., Di Pietro A. (2006). Purification of breast cancer resistance protein ABCG2 and role of arginine-482. Cell Mol. Life Sci..

[20] Zhou S., Schuetz J.D., Bunting K.D., Colapietro A.M., Sampath J., Morris J.J., Lagutina I., Grosveld G.C., Osawa M., Nakauchi H. (2001). The ABC transporter Bcrp1/ABCG2 is expressed in a wide variety of stem cells and is a molecular determinant of the side-population phenotype. Nat. Med..

[21] Alqawi O., Bates S., Georges E. (2004). Arginine482 to threonine mutation in the breast cancer resistance protein ABCG2 inhibits rhodamine 123 transport while increasing binding. Biochem. J..

[22] Gupta A., Dai Y., Vethanayagam R.R., Hebert M.F., Thummel K.E., Unadkat J.D., Ross D.D., Mao Q. (2006). Cyclosporin A, tacrolimus and sirolimus are potent inhibitors of the human breast cancer resistance protein (ABCG2) and reverse resistance to mitoxantrone and topotecan. Cancer Chemother. Pharmacol..

[23] Mao Q., Conseil G., Gupta A., Cole S.P., Unadkat J.D. (2004). Functional expression of the human breast cancer resistance protein in *Pichia pastoris*. Biochem. Biophys. Res. Commun..

[24] Ejendal K.F., Hrycyna C.A. (2005). Differential sensitivities of the human ATP-binding cassette transporters ABCG2 and P-glycoprotein to cyclosporin A. Mol. Pharmacol..

[25] Kannan P., Telu S., Shukla S., Ambudkar S.V., Pike V.W., Halldin C., Gottesman M.M., Innis R.B., Hall M.D. (2011). The “specific” P-glycoprotein inhibitor Tariquidar is also a substrate and an inhibitor for breast cancer resistance protein (BCRP/ABCG2). ACS Chem. Neurosci..

[26] Minderman H., Brooks T.A., O’Loughlin K.L., Ojima I., Bernacki R.J., Baer M.R. (2004). Broad-spectrum modulation of ATP-binding cassette transport proteins by the taxane derivatives ortataxel (IDN-5109, BAY 59-8862) and tRA96023. Cancer Chemother. Pharmacol..

[27] Ozvegy-Laczka C., Hegedus T., Várady G., Ujhelly O., Schuetz J.D., Váradi A., Kéri G., Orfi L., Német K., Sarkadi B. (2004). High-affinity interaction of tyrosine kinase inhibitors with the ABCG2 multidrug transporter. Mol. Pharmacol..

[28] Jacob L., Hoffmann B., Stoven V., Vert J.P. (2008). Virtual screening of GPCRs: An *in silico* chemogenomics approach. BMC Bioinform..

[29] Ding Y.L., Shih Y.H., Tsai F.Y., Leong M.K. (2014). *In silico* prediction of inhibition of promiscuous breast cancer resistance protein (BCRP/ABCG2). PLoS ONE.

[30] Villoutreix B.O., Kuenemann M.A., Poyet J.L., Bruzzoni-Giovanelli H., Labbé C., Lagorce D., Sperandio O., Miteva M.A. (2014). Drug-like protein—Protein interaction modulators: Challenges and opportunities for drug discovery and chemical biology. Mol. Inform..

[31] Hazai E., Bikádi Z. (2008). Homology modeling of breast cancer resistance protein (ABCG2). J. Struct. Biol..

[32] Polgar O., Ierano C., Tamaki A., Stanley B., Ward Y., Xia D., Tarasova N., Robey R.W., Bates S.E. (2010). Mutational analysis of threonine 402 adjacent to the GXXXG dimerization motif in transmembrane segment 1 of ABCG2. Biochemistry.

[33] Ni Z., Bikadi Z., Rosenberg M.F., Mao Q. (2010). Structure and function of the human breast cancer resistance protein (BCRP/ABCG2). Curr. Drug Metab..

[34] Li Y.F., Polgar O., Okada M., Esser L., Bates S.E., Xia D. (2007). Towards understanding the mechanism of action of the multidrug resistance-linked half-ABC transporter ABCG2: A molecular modeling study. J. Mol. Graph. Model..

[35] Guha R. (2013). On exploring structure-activity relationships. Methods Mol. Biol..

[36] Peltason L., Bajorath J. (2009). Systematic computational analysis of structure-activity relationships: Concepts, challenges and recent advances. Future Med. Chem..

[37] Erić S., Kalinić M., Ilić K., Zloh M. (2014). Computational classification models for predicting the interaction of drugs with P-glycoprotein and breast cancer resistance protein. SAR QSAR Environ. Res..

[38] Rangel L.P., Winter E., Gauthier C., Terreux R., Chiaradia-Delatorre L.D., Mascarello A., Nunes R.J., Yunes R.A., Creczynski-Pasa T.B., Macalou S. (2013). New structure-activity relationships of chalcone inhibitors of breast cancer resistance protein: Polyspecificity toward inhibition and critical substitutions against cytotoxicity. Drug Des. Devel. Ther..

[39] Han Y., Riwanto M., Go M.L., Ee P.L. (2008). Modulation of breast cancer resistance protein (BCRP/ABCG2) by non-basic chalcone analogues. Eur. J. Pharm. Sci..

[40] Liu X.L., Tee H.W., Go M.L. (2008). Functionalized chalcones as selective inhibitors of P-glycoprotein and breast cancer resistance protein. Bioorg. Med. Chem..

[41] Juvale K., Pape V.F., Wiese M. (2012). Investigation of chalcones and benzochalcones as inhibitors of breast cancer resistance protein. Bioorg. Med. Chem..

[42] Sim H.M., Lee C.Y., Ee P.L., Go M.L. (2008). Dimethoxyaurones: Potent inhibitors of ABCG2 (breast cancer resistance protein). Eur. J. Pharm. Sci..

[43] Boumendjel A., Nicolle E., Moraux T., Gerby B., Blanc M., Ronot X., Boutonnat J. (2005). Piperazinobenzopyranones and phenalkylaminobenzopyranones: Potent inhibitors of breast cancer resistance protein (ABCG2*)*. J. Med. Chem..

[44] Nicolle E., Boccard J., Guilet D., Dijoux-Franca M.G., Zelefac F., Macalou S., Grosselin J., Schmidt J., Carrupt P.A., di Pietro A. (2009). Breast cancer resistance protein (BCRP/ABCG2): New inhibitors and QSAR studies by a 3D linear solvation energy approach. Eur. J. Pharm. Sci..

[45] Valdameri G., Genoux-Bastide E., Peres B., Gauthier C., Guitton J., Terreux R., Winnischofer S.M., Rocha M.E., Boumendjel A., di Pietro A. (2012). Substituted chromones as highly potent nontoxic inhibitors, specific for the breast cancer resistance protein. J. Med. Chem..

[46] Valdameri G., Genoux-Bastide E., Gauthier C., Peres B., Terreux R., Winnischofer S.M., Rocha M.E., di Pietro A., Boumendjel A. (2012). 6-halogenochromones bearing tryptamine: One-step access to potent and highly selective inhibitors of breast cancer resistance protein. Chem. Med. Chem..

[47] Honorat M., Guitton J., Gauthier C., Bouard C., Lecerf-Schmidt F., Peres B., Terreux R., Gervot H., Rioufol C., Boumendjel A. (2014). MBL-II-141, a chromone derivative, enhances irinotecan (CPT-11) anticancer efficiency in ABCG2-positive xenografts. Oncotarget.

[48] Gerwick W.H., Moore B.S. (2012). Lessons from the past and charting the future of marine natural products drug discovery and chemical biology. Chem. Biol..

[49] Martins A., Vieira H., Gaspar H., Santos S. (2014). Marketed marine natural products in the pharmaceutical and cosmeceutical industries: Tips for success. Mar. Drugs.

[50] Rocha-Martin J., Harrington C., Dobson A.D., O’Gara F. (2014). Emerging strategies and integrated systems microbiology technologies for biodiscovery of marine bioactive compounds. Mar. Drugs.

[51] Siegel R., DeSantis C., Virgo K., Stein K., Mariotto A., Smith T., Cooper D., Gansler T., Lerro C., Fedewa S. (2012). Cancer treatment and survivorship statistics, 2012. CA Cancer. J. Clin..

[52] Saraswathy M., Gong S. (2013). Different strategies to overcome multidrug resistance in cancer. Biotechnol. Adv..

[53] Kibria G., Hatakeyama H., Harashima H. (2014). Cancer multidrug resistance: Mechanisms involved and strategies for circumvention using a drug delivery system. Arch. Pharm. Res..

[54] Lopez D., Martinez-Luis S. (2014). Marine natural products with P-glycoprotein inhibitor properties. Mar. Drugs.

[55] Wang F., Fang Y., Zhu T., Zhang M., Lin A., Gu Q., Zhu W. (2008). Seven new prenylated indole diketopiperazine alkaloids from holothurian-derived fungus *Aspergillus fumigatus*. Tetrahedron.

[56] Wang Y., Li Z.L., Bai J., Zhang L.M., Wu X., Zhang L., Pei Y.H., Jing Y.K., Hua H.M. (2012). 2,5-diketopiperazines from the marine-derived fungus *Aspergillus fumigatus* YK-7. Chem. Biodivers..

[57] Zhang M., Wang W.L., Fang Y.C., Zhu T.J., Gu Q.Q., Zhu W.M. (2008). Cytotoxic alkaloids and antibiotic nordammarane triterpenoids from the marine-derived fungus Aspergillus sydowi. J. Nat. Prod..

[58] Zhao W.Y., Zhang Y.P., Zhu T.J., Fang Y.C., Liu H.B., Gu Q.Q., Zhu W.M. (2006). Studies on the indolyl diketopiperazine analogs produced by marine-derived fungus A-f-11. Chin. J. Antibiot..

[59] Rabindran S.K., He H., Singh M., Brown E., Collins K.I., Annable T., Greenberger L.M. (1998). Reversal of a novel multidrug resistance mechanism in human colon carcinoma cells by fumitremorgin C. Cancer Res..

[60] Hazlehurst L.A., Foley N.E., Gleason-Guzman M.C., Hacker M.P., Cress A.E., Greenberger L.W., de Jong M.C., Dalton W.S. (1999). Multiple mechanisms confer drug resistance to mitoxantrone in the human 8226 myeloma cell line. Cancer Res..

[61] Rabindran S.K., Ross D.D., Doyle L.A., Yang W., Greenberger L.M. (2000). Fumitremorgin C reverses multidrug resistance in cells transfected with the breast cancer resistance protein. Cancer Res..

[62] Plate R., Hermkens P.H.H., Behm H., Ottenheijm H.C.J. (1987). Application of an isoxazolidine in a stereoselective approach to the fumitremorgin series. J. Org. Chem..

[63] Van Loevezijn A., Allen J.D., Schinkel A.H., Koomen G.J. (2001). Inhibition of BCRP-mediated drug efflux by fumitremorgin-type indolyl diketopiperazines. Bioorg. Med. Chem. Lett..

[64] Van Maarseveen J.H. (1998). Solid phase synthesis of heterocycles by cyclization/cleavage methodologies. Comb. Chem. High Throughput Screen..

[65] Allen J.D., van Loevezijn A., Lakhai J.M., van der Valk M., van Tellingen O., Reid G., Schellens J.H., Koomen G.J., Schinkel A.H. (2002). Potent and specific inhibition of the breast cancer resistance protein multidrug transporter *in vitro* and in mouse intestine by a novel analogue of fumitremorgin C. Mol. Cancer Ther..

[66] Cui C.B., Kakeya H., Okada G., Onose R., Ubukata M., Takahashi I., Isono K., Osada H. (1995). Tryprostatins A and B, novel mammalian cell cycle inhibitors produced by *Aspergillus fumigatus*. J. Antibiot..

[67] Cui C.B., Kakeya H., Okada G., Onose R., Osada H. (1996). Novel mammalian cell cycle inhibitors, tryprostatins A, B and other diketopiperazines produced by *Aspergillus fumigatus*. I. Taxonomy, fermentation, isolation and biological properties. J. Antibiot..

[68] Cui C.B., Kakeya H., Osada H. (1996). Novel mammalian cell cycle inhibitors, tryprostatins A, B and other diketopiperazines produced by *Aspergillus fumigatus*. II. Physico-chemical properties and structures. J. Antibiot..

[69] Woehlecke H., Osada H., Herrmann A., Lage H. (2003). Reversal of breast cancer resistance protein-mediated drug resistance by tryprostatin A. Int. J. Cancer.

[70] Patel K., Gadewar M., Tripathi R., Prasad S.K., Patel D.K. (2012). A review on medicinal importance, pharmacological activity and bioanalytical aspects of beta-carboline alkaloid “Harmine”. Asian Pac. J. Trop. Biomed..

[71] Khan A.M., Noreen S., Imran Z.P., Choudhary M.I. (2011). A new compound, jolynamine, from marine brown alga *Jolyna laminarioides*. Nat. Prod. Res..

[72] Zaker F., Oody A., Arjmand A. (2007). A study on the antitumoral and differentiation effects of Peganum harmala derivatives in combination with ATRA on leukaemic cells. Arch. Pharm. Res..

[73] Ma Y., Wink M. (2010). The beta-carboline alkaloid harmine inhibits BCRP and can reverse resistance to the anticancer drugs mitoxantrone and camptothecin in breast cancer cells. Phytother. Res..

[74] McDonald L.A., Swersey J.C., Ireland C.M., Carroll A.R., Coll J.C., Bowden B.F., Fairchild C.R., Cornell L. (1995). Botryllamides A-D, new brominated tyrosine derivatives from styelid ascidians of the genus *Botryllus*. Tetrahedron.

[75] Rao M.R., Faulkner D.J. (2004). Botryllamides E–H, four new tyrosine derivatives from the ascidian *Botrylloides tyreum*. J. Nat. Prod..

[76] McKay M.J., Carroll A.R., Quinn R.J. (2005). Perspicamides A and B, quinolinecarboxylic acid derivatives from the Australian ascidian *Botrylloides perspicuum*. J. Nat. Prod..

[77] Henrich C.J., Robey R., Takada K., Bokesch H.R., Bates S.E., Shukla S., Ambudkar S.V., McMahon J.B., Gustafson K.R. (2009). Botryllamides: Natural product inhibitors of ABCG2. ACS Chem. Biol..

[78] Takada K., Imamura N., Gustafson K.R., Henrich C.J. (2010). Synthesis and structure-activity relationship of botryllamides that block the ABCG2 multidrug transporter. Bioorg. Med. Chem. Lett..

[79] Andersen R.J., Faulkner D.J., He C.H., van Duyne G.D., Clardy J. (1985). Metabolites of the marine prosobranch mollusk *Lamellaria* sp.. J. Am. Chem. Soc..

[80] Lindquist N., Fenical W., van Duyne G.D., Clardy J. (1988). New alkaloids of the lamellarin class from the marine ascidian *Didemnum chartaceum* (Sluiter, 1909). J. Org. Chem..

[81] Davis R.A., Carroll A.R., Pierens G.K., Quinn R.J. (1999). New lamellarin alkaloids from the Australian ascidian, *Didemnum chartaceum*. J. Nat. Prod..

[82] Urban S., Butler M.S., Capon R.J. (1994). Lamellarins O and P: New aromatic metabolites from the Australian marine sponge *Dendrilla cactos*. Aust. J. Chem..

[83] Urban S., Hobbs L., Hooper J.N.A., Capon R.J. (1995). Lamellarins Q and R: New aromatic metabolites from an Australian marine sponge *Dendrilla cactos*. Aust. J. Chem..

[84] Carroll A.R., Bowden B.F., Coll J.C. (1993). Studies of Australian ascidians. I. Six new lamellarin-class alkaloids from a colonial ascidian, *Didemnum* sp.. Austr. J. Chem..

[85] Urban S., Capon R.J. (1996). Lamellarin S: A new aromatic metabolite from an Australian tunicate *Didemnum* sp.. Aust. J. Chem..

[86] Reddy M.V.R., Faulkner D.J., Venkateswarlu Y., Rao M.R. (1997). New lamellarin alkaloids from an unidentified ascidian from the Arabian Sea. Tetrahedron.

[87] Bailly C. (2004). Lamellarins, from A to Z: A family of anticancer marine pyrrole alkaloids. Curr. Med. Chem. Anticancer Agents.

[88] Huang X.C., Xiao X., Zhang Y.K., Talele T.T., Salim A.A., Chen Z.S., Capon R.J. (2014). Lamellarin O, a pyrrole alkaloid from an Australian marine sponge, *Ianthella* sp., reverses BCRP mediated drug resistance in cancer cells. Mar. Drugs.

[89] Tsubotani S.H., Tanida S., Harada S. (1991). Structure determination of indolocarbazole alkaloids by NMR spectroscopy. Tetrahedron.

[90] Sánchez C., Zhu L., Braña A.F., Salas A.P., Rohr J., Méndez C., Salas J.A. (2005). Combinatorial biosynthesis of antitumor indolocarbazole compounds. Proc. Natl. Acad. Sci. USA.

[91] Sánchez C., Méndez C., Salas J.A. (2006). Indolocarbazole natural products: Occurrence, biosynthesis, and biological activity. Nat. Prod. Rep..

[92] Liu R., Zhu T., Li D., Gu J., Xia W., Fang Y., Hongbing L., Zhu W., Gu Q. (2007). Two indolocarbazole alkaloids with apoptosis activity from a marine-derived actinomycete Z(2)039-2. Arch. Pharm. Res..

[93] Robey R.W., Shukla S., Steadman K., Obrzut T., Finley E.M., Ambudkar S.V., Bates S.E. (2007). Inhibition of ABCG2-mediated transport by protein kinase inhibitors with a bisindolylmaleimide or indolocarbazole structure. Mol. Cancer Ther..

[94] Yin Y., Xu B., Li Z., Zhang B. (2013). Enhanced production of (+)-terrein in fed-batch cultivation of *Aspergillus terreus* strain PF26 with sodium citrate. World J. Microbiol. Biotechnol..

[95] Wang Y., Zheng J., Liu P., Wang W., Zhu W. (2011). Three new compounds from *Aspergillus terreus* PT06–2 grown in a high salt medium. Mar. Drugs.

[96] Malmstrøm J., Christophersen C., Barrero A.F., Oltra J.E., Justicia J., Rosales A. (2002). Bioactive metabolites from a marine-derived strain of the fungus *Emericella variecolor*. J. Nat. Prod..

[97] Kim D.S., Cho H.J., Lee H.K., Lee W.H., Park E.S., Youn S.W., Park K.C. (2007). Terrein, a fungal metabolite, inhibits the epidermal proliferation of skin equivalents. J. Dermatol. Sci..

[98] Phattanawasin P., Pojchanakom K., Sotanaphun U., Piyapolrungroj N., Zungsontiporn S. (2007). Weed growth inhibitors from *Aspergillus fischeri* TISTR 3272. Nat. Prod. Res..

[99] Lee J.C., Yu M.K., Lee R., Lee Y.H., Jeon J.G., Lee M.H., Jhee E.C., Yoo I.D., Yi H.K. (2008). Terrein reduces pulpal inflammation in human dental pulp cells. J. Endodont..

[100] Arakawa M., Someno T., Kawada M., Ikeda D. (2008). A new terrain glucoside, a novel inhibitor of angiogenin secretion in tumor angiogenesis. J. Antibiot..

[101] Lee Y.H., Lee N.H., Bhattarai G., Oh Y.T., Yu M.K., Yoo I.D., Jhee E.C., Yi H.K. (2010). Enhancement of osteoblast biocompatibility on titanium surface with terrein treatment. Cell Biochem. Funct..

[102] Liao W.Y., Shen C.N., Lin L.H., Yang Y.L., Han H.Y., Chen J.W., Kuo S.C., Wu S.H., Liaw C.C. (2012). Asperjinone, a nor-neolignan, and terrein, a suppressor of ABCG2-expressing breast cancer cells, from thermophilic *Aspergillus terreus*. J. Nat. Prod..

[103] Steyn P.S. (1970). The isolation, structure and absolute configuration of secalonic acid D, the toxic metabolite of *Penicillium oxalicum*. Tetrahedron.

[104] Zhang J.Y., Tao L.Y., Liang Y.J., Yan Y.Y., Dai C.L., Xia X.K., She Z.G., Lin Y.Ch., Fu L.W. (2009). Secalonic acid D induced leukemia cell apoptosis and cell cycle arrest of G1 with involvement of GSK-3β/β-catenin/c-Myc pathway. Cell Cycle.

[105] Guo Z., Shao C., She Z., Cai X., Liu F., Vrijimoed L.L., Lin Y. (2007). 1H and 13C NMR assignments for two oxaphenalenones bacillosporin C and D from the mangrove endophytic fungus SBE-14. Magn. Reson. Chem..

[106] Ren H., Tian L., Gu Q., Zhu W. (2006). Secalonic acid D; A cytotoxic constituent from marine lichen-derived fungus *Gliocladium* sp. T31. Arch. Pharm. Res..

[107] Hong R. (2011). Secalonic acid D as a novel DNA topoisomerase I inhibitor from marine lichen-derived fungus *Gliocladium* sp. T31. Pharm. Biol..

[108] Hu Y.P., Tao L.Y., Wang F., Zhang J.Y., Liang Y.J., Fu L.W. (2013). Secalonic acid D reduced the percentage of side populations by down-regulating the expression of ABCG2. Biochem. Pharmacol..

[109] Bokesch H.R., Cartner L.K., Fuller R.W., Wilson J.A., Henrich C.J., Kelley J.A., Gustafson K.R., McMahon J.B., McKee T.C. (2010). Inhibition of ABCG2-mediated drug efflux by naphthopyrones from marine crinoids. Bioorg. Med. Chem. Lett..

